# Automatic and Robust Infrared-Visible Image Sequence Registration via Spatio-Temporal Association

**DOI:** 10.3390/s19050997

**Published:** 2019-02-26

**Authors:** Bingqing Zhao, Tingfa Xu, Yiwen Chen, Tianhao Li, Xueyuan Sun

**Affiliations:** 1 Image Engineering & Video Technology Lab, School of Optics and Photonics, Beijing Institute of Technology, Beijing 100081, China; zhaobq94@163.com (B.Z.); cyw951025@163.com (Y.C.); 2120170544@bit.edu.cn (T.L.); 2220170305@bit.edu.cn (X.S.); 2 Key Laboratory of Photoelectronic Imaging Technology and System, Ministry of Education of China, Beijing 100081, China

**Keywords:** image registration, temporal motion information, foreground contour, FAST corner, spatial location distribution, reservoir

## Abstract

To solve the problems of the large differences in gray value and inaccurate positioning of feature information during infrared-visible image registration, we propose an automatic and robust algorithm for registering planar infrared-visible image sequences through spatio-temporal association. In particular, we first create motion vector distribution descriptors which represent the temporal motion information of foreground contours in adjacent frames to complete coarse registration without feature extraction. Then, for precise registration, we extracted FAST corners of the foreground, which are described by the spatial location distribution of contour points based on connected blob detection, and match these corners using bidirectional optimal maximum strategy. Finally, a reservoir updated by Better-In, Worse-Out (BIWO) strategy is established to save matched point pairs and obtain the optimal global transformation matrix. Extensive evaluations on the LITIV dataset well demonstrate the effectiveness of the proposed algorithm. Particularly, our algorithm achieves lower registration overlapping errors than the other two state-of-the-arts.

## 1. Introduction

Multi-sensor image fusion [1,2,3,4], which can enhance the ability of target description and scene understanding, is widely used in target tracking [5], face recognition [6], night vision observation [7] and many other fields. Image registration, as an important procedure for image fusion, greatly determines the accuracy of target alignment in the scene, thus affecting the quality of infrared-visible image fusion. 

There exist two challenging problems in infrared-visible image registration. On the one hand, images from different sensors manifest different phenomena [8]. Visible images record the reflected light information of objects while infrared images record the thermal field distribution. Thus, the gray value of two types of images are quite different. On the other hand, directly extracting feature information across different types of images for registration [8,9,10] may lead to inaccurate positioning, which would reduce the accuracy of the final transformation matrix for alignment. 

To overcome the challenges above, many effective algorithms [11,12,13,14,15] have been proposed in recent years. Most of them are committed to using motion information to register targets in the scene. St. Charles et al. [14] proposed a framework based on shape matching, but its performance depends largely on foreground detection. Sun et al. [15] proposed combining motion and feature information to register targets. However, it cannot provide satisfactory results during special moments.

We propose a coarse-to-precise registration algorithm for infrared-visible image sequences via spatio-temporal association. In the coarse registration step, the motion vector field of the detected foreground is calculated. Inspired by the Histograms of Oriented Gradients (HOG) [16], we first create Motion Vector Distribution (MVD) descriptors to represent temporal motion information of foreground contours, and then randomly select contour points in the infrared image and search for their corresponding contour points in the visible image without feature extraction. Such coarse registration can roughly align targets in the scene and thus eliminate the influence of inaccurate positioning of feature information. In the precise registration step which is based on intra-frame feature matching, we first relocate foregrounds with the computed coarse parameters, and then extract FAST corners [17] of the targets. We further use shape context descriptors [18] to describe the spatial location distribution of the connected blob contours. To match these feature points, we create bidirectional optimal maximum strategy and establish a reservoir updated by Better-In, Worse-out (BIWO) strategy to save them. The optimal global transformation matrix is computed using the matched point pairs in the reservoir. Benefiting from the use of temporal motion information, stricter feature description and matching, and a robust reservoir, the proposed algorithm can achieve high registration accuracy. To sum up, the main contributions of this work are as follows:

(1) We propose a spatio-temporal associated registration algorithm for infrared-visible image sequences, which combines temporal motion information and intra-frame feature matching scheme, achieving low registration overlapping errors.
(2) We create MVD descriptors of foreground contours for coarse registration without feature extraction. Thus, foreground targets can be roughly aligned to eliminate the impact of inaccurate positioning of feature points.
(3) We propose a description of feature points based on the spatial location distribution of connected blob contours, and perform feature matching using bidirectional optimal maximum strategy. A robust reservoir updated by BIWO strategy is proposed to improve the accuracy of the final global transformation matrix.

The rest of the paper is structured as follows: Section 2 provides a review of related work. Section 3 introduces algorithm overview and details of each procedure. Section 4 presents qualitative and quantitative evaluations of the proposed algorithm as well as comparisons with other state-of-the-arts. Section 5 presents the conclusions.

## 2. Related Work

Methods for image registration can be categorized into two types: intensity-based methods and feature-based methods. Intensity-based registration methods use the gray values of all pixels to calculate the similarity between two images, and adopt search methods such as genetic algorithm [19], ant colony algorithm [20], Powell algorithm [21], etc. to get the optimal transformation matrix. Cross-correlation methods [22] use the regional cross-correlation between two images as the similarity measure function for registration. However, the computational complexity of such methods is quite high. Mutual information methods [21,23] utilize the statistical correlation between images for registration, which are commonly used in multimodal medical image registration. Phase correlation methods [24,25] are effective complements to image registration, which compute the parameters of scaling, rotation and translation by calculating the maximum cross power spectrum of two images from frequency domain. Intensity-based registration methods can hardly be applied in infrared-visible images due to the huge difference in gray value. Furthermore, textures in visible images are often missed in infrared images as they seldom influence heat emitted by targets.

Feature-based registration methods extract feature information such as points [9,10], edges [26], contours [8,14], etc. of the images, which are robust to illumination, temperature, occlusion and many other disruptive factors. It is unreliable to directly use edges or contours as they are not completely consistent across infrared and visible images. Points are the simplest but most efficient feature information and FAST corner [17] has developed rapidly in computer vision tasks for its fast computing speed. Similarly, when there exists large spatial variation between infrared and visible images, the positioning of feature points will have a certain deviation, which means that methods of directly using feature points for registration [9,10] are inapplicable to our task. 

Registration of infrared-visible image sequence has attracted much attention in recent years. Image sequence can provide motion information between adjacent frames, which can be complementary to image registration. Methods based on global optical flow information [11] need to calculate the optical flow of all frames, making their use impossible for real-time registration. Methods based on target trajectories were proposed in [12,13]. The positioning of the centroids or apexes of the targets may not be accurate enough, and thus trajectories formed by these points may not fully reflect the motion state of the targets. Sun et al. [15] proposed to combine motion vectors and feature information. However, the calculation of motion vectors requires a consistent number of targets in the scene so that such method is not suitable for situations in which targets enter or leave the scene. In the proposed registration algorithm, the problem of inaccurate positioning of feature points is overcome by coarsely registering foreground contours using MVD descriptors. We create a strict matching strategy as well as a robust reservoir to match and save point pairs extracted from relocated foregrounds, and to calculate the optimal global transformation matrix. Main procedures will be detailed below.

## 3. Methodology

### 3.1. Overview of the Proposed Algorithm

**Figure 1:**
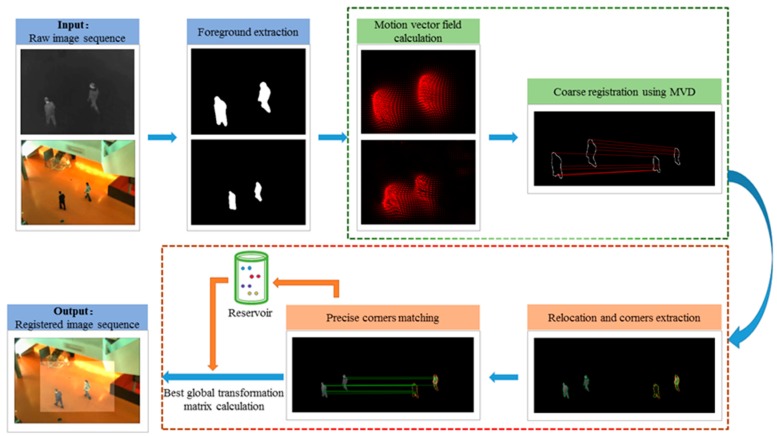
Flow chart of the proposed algorithm.

Moving targets in the scene can provide available motion information. We extract the foregrounds of the images and accomplish registration on them. Figure 1 shows an overall flow of the proposed registration algorithm for infrared-visible image sequences. It mainly consists of three procedures: foreground extraction, coarse registration based on temporal motion information, and precise registration based on intra-frame feature matching.

In coarse registration, we calculate and rectify the motion vector field of two adjacent frames. The motion vectors on the foreground contours can fully reflect the motion state of the target. Motion vectors of different parts and different targets are discriminative. Inspired by the HOG [16], we create MVD descriptors for foreground contours and use corresponding foreground contour points to calculate coarse parameters of the transformation matrix.

In precise registration, FAST corners [17] of the relocated foregrounds are extracted. These points are then described by the shape context descriptors [18] of the connected blob contours to which they belong and matched by bidirectional optimal maximum strategy. A reservoir based on BIWO update strategy is established to save matched point pairs and to calculate precise parameters of the transformation matrix.

It should be noted that infrared image is the floating image while visible image is the reference image in the proposed algorithm. The transformation model we use is similar transformation matrix including scaling, rotation and translations, which is:

(1) $$\left[\begin{array}{c} y^{IR}\\ x^{IR}\\ 1 \end{array}\right]=\left[\begin{array}{ccc} \sigma \cos\theta & \sigma \sin\theta & \Delta y\\ -\sigma \sin\theta & \sigma \cos\theta & \Delta x\\ 0 & 0 & 1 \end{array}\right]\left[\begin{array}{c} y^{VIS}\\ x^{VIS}\\ 1 \end{array}\right]$$

where $\left(x^{IR},y^{IR}\right)$ and $\left(x^{VIS},y^{VIS}\right)$ are the positions of pixels in infrared and visible images, respectively. σ is the scaling factor, θ is the rotation factor, and Δy, Δ*x* are the vertical and horizontal translation factors. The proposed algorithm is focused on registering planar scenes, which means that various sensors capture images at long distance from targets.

### 3.2. Foreground Extraction

To fully make use of the temporal motion information, we perform foreground extraction by using the method in [27]. It uses color and texture information to define the pixels as “background words” in local dictionaries and introduce a feedback mechanism to continuously upgrade the model. In the subsequent coarse registration process, we use the motion vector field of the foreground. And in the precise registration process, we retain FAST corners of the relocated foreground.

### 3.3. Coarse Registration 

When the spatial transformation of two images differs greatly, direct extraction of feature points may result in inaccurate positioning. Coarse registration on foreground contours can solve this problem, which we will explain in detail.

#### 3.3.1. Image Preprocessing

We perform some preprocessing on the images to obtain a more accurate motion vector field:

- The lighting condition may change when sensors capture images, which will greatly affect the accuracy of the motion vector field. For an image sequence with a resolution of *M* × *N*, the gray value of the pixels in the next frame $g_{t+1}(i,j)$ will be rectified to the previous frame $g_{t}(i,j)$ by:
  (2) $$g_{t+1}{\left(i,j\right)}^{\prime }=g_{t+1}(i,j)\times \left[\frac{1}{M\times N}\sum_{i=1}^{M}\sum_{j=1}^{N}g_{t}(i,j)/\frac{1}{M\times N}\sum_{i=1}^{M}\sum_{j=1}^{N}g_{t+1}(i,j)\right]$$
- Noise removal is necessary. We use a Gaussian filter (5 × 5 size, standard deviation of 3) to smooth each frame of the image sequence.

#### 3.3.2. Motion Vector Field Calculation

**Figure 2:**
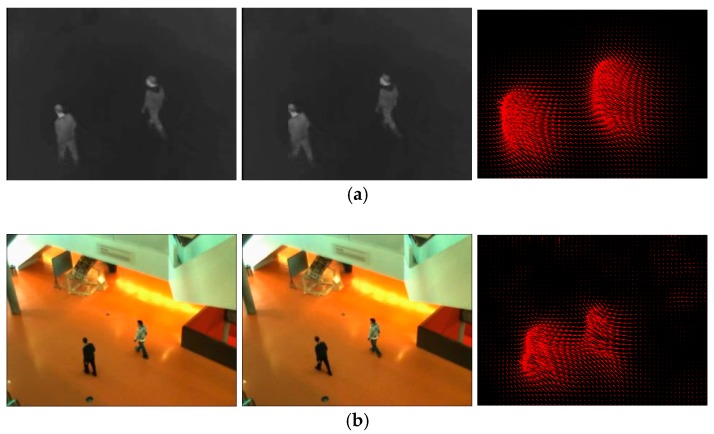
Adjacent frames (**left** and **middle**) and the motion vector diagrams (**right**) with display step size of 5 pixels. The red arrow direction represents the motion vector direction of the pixel, and the arrow length represents the normalized motion vector magnitude. (**a**) Infrared image; (**b**) Visible image.

To obtain motion vector field of the whole image, we calculate optical flow which represents the instantaneous motion velocity of each pixel. [28,29] are typical methods for optical flow calculation. But they are inapplicable when the speed of moving targets is too low or too fast, especially in our task where sensors capture images far from moving targets in the scene. We adopt an improved optical flow calculation method presented in [30]. It defines an improved variational equation and introduces a smoothness constraint to minimize it. Figure 2 shows the motion vector diagrams obtained by adjacent frames in the image sequence.

#### 3.3.3. Motion Vector Field Filtering and Re-Projection

- Motion vector that belongs to the background is set to zero. Because only the motion vector of the foreground is sufficiently distinguishable for registering foreground contours.
- Motion vector near the image boundaries tends to be inaccurate and is not conducive to the establishment of subsequent MVD descriptors. We remove the motion vector near the boundaries with a threshold of 20 pixels.
- For a pixel with location (x,y), gray value $g_{t}$, and calculated motion vector $u=(u_{x},u_{y})$, the offset of the gray value relative to the pixel in the next frame $g_{t+1}$ can be obtained by re-projection (bilinear interpolation method):
  (3) $$\Delta g=\left|g_{t+1}(x+u_{x},y+u_{y})-g_{t}\right|$$

If the offset is greater than the threshold (5 pixels in our algorithm), motion vector of this pixel is considered to be wrong and is set to zero. 

#### 3.3.4. Creation of Motion Vector Distribution Descriptor and Contour Matching

For a pixel with $u=(u_{x},u_{y})$, we can get magnitude and orientation of motion vector by:

(4) $$\left\{ \begin{array}{l} Mag=\sqrt{{u_{x}}^{2}+{u_{y}}^{2}}\\ Ori=\arctan[u_{x}/(u_{y}+eps)]\in [0,2\pi ) \end{array}\right.$$

where eps is the minimum floating point precision, ensuring the denominator is not zero. Magnitude of motion vector has rotation and translation invariance. To obtain scaling invariance, for all motion vector magnitudes Mag(i,j), we normalize and encode them by:

(5) $$Mag^{\prime }(i,j)=\{\begin{array}{cc} 0 & if\;Mag(i,j)=0\\ round[Mag(i,j)/(Mag_{\max}+eps)\times 36+0.5] & \mathrm{otherwise} \end{array}$$

where $Mag_{\max}$ is the maximum magnitude value.

Orientation of motion vector has scaling and translation invariance, but no rotation invariance. For all motion vector orientations Ori(i,j), we first encode them by:

(6) $$Ori^{\prime }(i,j)=\{\begin{array}{cc} 0 & if\;Mag(i,j)=0\\ round[Ori(i,j)/(2\pi +eps)\times 36+0.5] & \mathrm{otherwise} \end{array}$$

where each code denotes an interval of 10° and code 0 represents that magnitude value is zero.

To obtain rotation invariance, we then turn the main orientation, selected by the maximum in its statistical histogram, as code 1. The remaining orientations are recorded in a clockwise direction as code 2 to 36:

(7) $$Ori^{\prime \prime }(i,j)=\{\begin{array}{cc} 0 & if\;Ori^{\prime }(i,j)=0\\ 36 & if\;\mathrm{mod}[Ori^{\prime }(i,j)-M^{code}+1.36]=0\\ \mathrm{mod}[Ori^{\prime }(i,j)-M^{code}+1.36] & \mathrm{otherwise} \end{array}$$

where $M^{code}$ is the main orientation code before recording.

Inspired by the HOG [16], taking a point on the foreground contour as the center, we first perform histogram statistics on non-zero magnitude and orientation of motion vector in a 9 × 9 pixels block. We then assign two-dimensional Gaussian distribution weight (9 × 9 size, standard deviation of 5) to generate it. We finally obtain encoded motion vector magnitude distribution vector $H^{Mag}$ and orientation distribution vector $H^{Ori}$ of the center point and both of them are 36-dimensional. Massive experiments demonstrate that under the planar condition, the speed of moving target in the scene is low, resulting in a smaller discrimination of motion vector magnitude distribution. When there are multiple targets moving in different directions in the scene, or when movements such as waving, turning, etc. occur, the distribution of motion vector orientation is more differentiated. Based on this, we add weight factor ω (ω=0.2 in our algorithm) and combine these two histogram vectors to create motion vector distribution descriptors (72-dimensional) for foreground contour points (the total number is *N*):

(8) $$MVD_{k}=[\omega H_{k}^{Mag}\;(1-\omega )H_{k}^{Ori}]\;k=1,2,\;\ldots ,\;N$$

**Figure 3:**
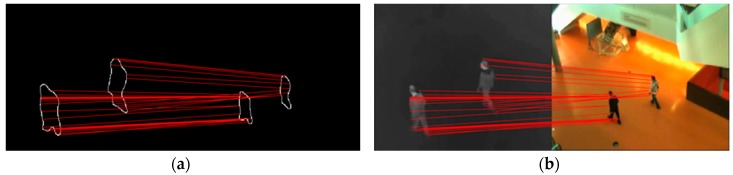
Matching result in the foreground contours (**a**) and in the original images (**b**) by using MVD descriptor. For the convenience of display, we only select 30 point pairs.

We randomly select some foreground contour points (in our algorithm, 10% of infrared contour point set each frame). For a selected infrared point, visible contour point with the smallest Euclidean distance between motion vector distribution descriptors is regarded as the corresponding point. The random sample consensus (RANSAC) algorithm [31] is adopted to calculate the optimal transformation matrix. Figure 3 shows the matching result of randomly selected foreground contour points.

There are some mismatches and inaccurate matched pairs, especially when targets in the scene are moving at a slower speed and the directions are almost consistent. RANSAC algorithm is able to calculate the optimal transformation matrix among all matched point pairs. Through coarse registration process, we can get coarse parameters of similarity transformation model: $\sigma^{c}$, $\theta^{c}$, $\Delta y^{c}$ and $\Delta x^{c}$.

### 3.4. Precise Registration 

Coarse registration cannot obtain accurate registration parameters due to its randomness. Importantly, it can roughly align targets in the scene for more accurate positioning of feature points. In the following section, the proposed precise registration is introduced.

#### 3.4.1. Relocation and Feature Point Extraction

**Figure 4:**
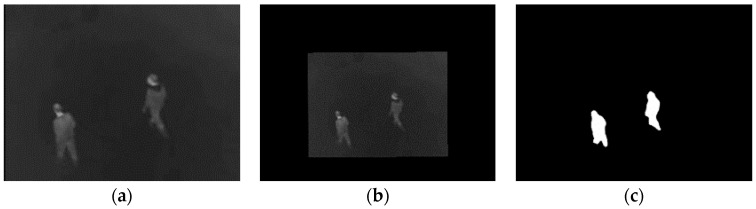
Original image (**a**) after relocation (**b**) and its relocated foreground (**c**).

We first relocate the floating image and foreground with coarse parameters (bilinear interpolation method). Figure 4 shows the results of relocated original image and foreground.

**Figure 5:**
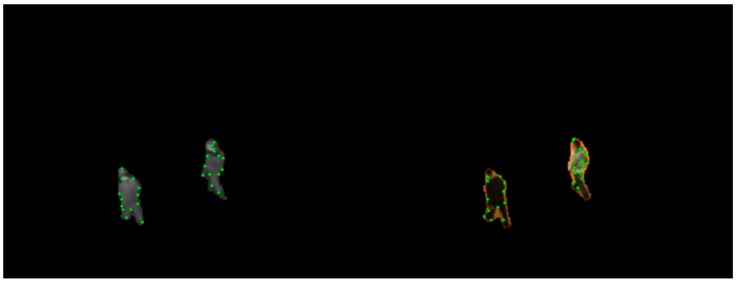
FAST corners detected in relocated infrared foreground (**left**) and visible foreground (**right**).

Since FAST corner detection algorithm [17] has low computational complexity and ensures accurate positioning, we choose to extract FAST corners of moving targets and match them. Similar to motion vector field filtering in coarse registration, corners that belong to the background and near the image boundaries are abandoned. Figure 5 shows the detected FAST corners.

#### 3.4.2. Feature Points Description

**Figure 6:**
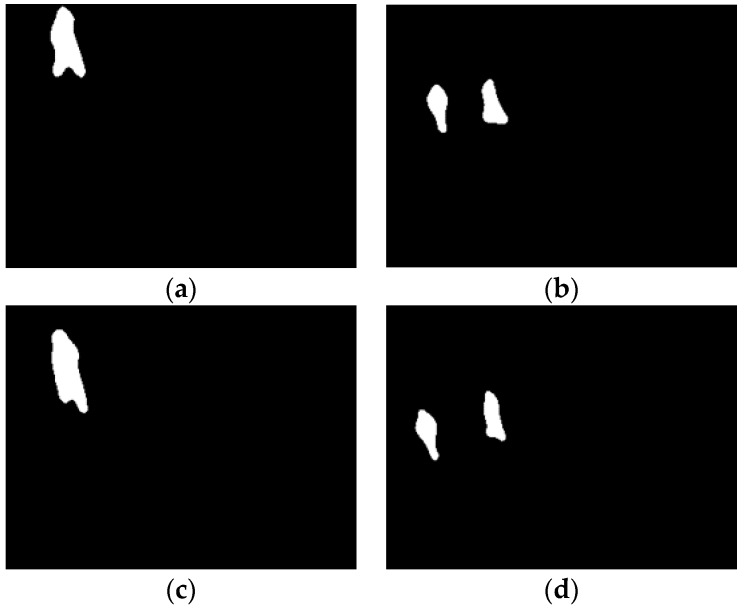
Targets enter (**a**,**b**) and leave (**c**,**d**) the scenes of infrared (**a**,**c**) and visible (**b**,**d**), respectively, at the same time.

In Reference [15], the number of targets in the scene needs to be consistent for homologous and heterogeneous feature point matching. Besides, in the process of normalized location description establishment, the centroid is obtained with all foregrounds. However, when moving targets just enter or leave the scene, the number of them tends to be inconsistent. As shown in Figure 6, there is one target entering or leaving the scene in the infrared while there are two in the visible. Reference [15] is unable to deal with these specific moments and the centroid calculation is inaccurate.

To register these special frames, we first use the two-pass algorithm to segment targets (foregrounds) in the scene, and merge the nearby small connected blobs caused by foreground extraction. In subsequent description, calculation of the centroid and establishment of the shape context descriptor are only performed in the connected foreground blob to which the feature point belongs. We then describe the feature point as follows:

- Position of the feature point: P=[x,y].
- Location of the feature point relative to the centroid of the connected foreground blob to which it belongs, calculated by:
  (9) $$L=[x-x^{c},y-y^{c}]$$
  where $\left[x^{c},y^{c}\right]$ is the position of the centroid.
- The shape context descriptor [18] of the feature point. It reflects the spatial location distribution of neighbored points around the center. Contour points of the connected foreground blob to which the feature point belongs form the descriptor. In our algorithm, log-polar coordinate is used to divide the distance into 5 bins and the angle into 8 bins. The shape context descriptor (40-dimensional) of the feature point is established by:
  (10) SC=[sc(1,1),sc(1,2),…,sc(dis,ang)] dis∈[1,5],ang∈[1,8]
  where sc(dis,ang) is the distribution statistical histogram of joint distance and angle.

#### 3.4.3. Matching

Three similarity metrics are used for feature point matching:

- Euclidean distance between positions of the two feature points:
  (11) $$S_{P}=\left|P^{IR}-P^{VIS}\right|$$
- Euclidean distance between locations of the two feature points relative to the centroids:
  (12) $$S_{L}=\left|L^{IR}-L^{VIS}\right|$$
- Chi-square test statistic between two shape context descriptors:
  (13) $$Cs=\frac{1}{2}\sum_{k=1}^{K}\frac{{\left[SC^{IR}(k)-SC^{VIS}(k)\right]}^{2}}{SC^{IR}(k)+SC^{VIS}(k)},\;K=40$$

Since coarse registration process has roughly aligned moving targets in the scene, we first treat a point pair as potentially matched if $S_{P}<S_{th1}$ and $S_{L}<S_{th2}$; otherwise we just ignore it and consider another point pair. Then we calculate chi-square test statistic between two shape descriptors. For an infrared feature point, visible feature point with the smallest chi-square test statistic is regarded as its matched point.

**Figure 7:**
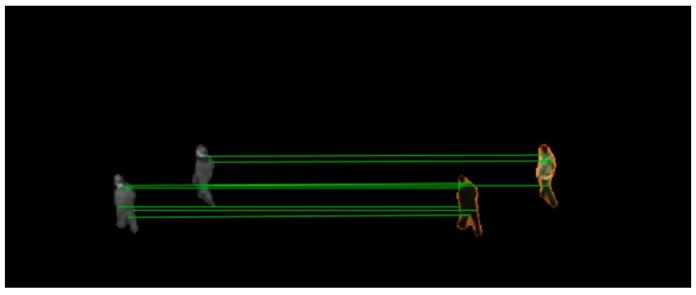
Matched point pairs by using bidirectional optimal maximum strategy.

There may come situations in which more than one visible feature point is matched to the same infrared feature point or mismatches happens. To solve them, as introduced in Algorithm 1, we create bidirectional optimal maximum strategy to filter the point pairs. Figure 7 shows the matched point pairs by using the proposed matching strategy.

**Algorithm 1:** Bidirectional Optimal Maximum Matching Strategy**Input:** Point sets $\left\{FP^{IR}\right\}$ and $\left\{FP^{VIS}\right\}$; descriptions $P^{IR}$, $L^{IR}$, $SC^{IR}$ and $P^{VIS}$, $L^{VIS}$, $SC^{VIS}$.**Output:** Matched point set {MP}.For each point in $\left\{FP^{IR}\right\}$
**Foreach**
$\left\{FP^{VIS}\right\}$
**If**$S_{P}<S_{th1}$ ($S_{th1}=20$ in our algorithm) & $S_{L}<S_{th2}$ ($S_{th2}=10$ in our algorithm)  Calculate Cs using Equation (13); get the minimun $Cs_{\min}$ and sub-minimum $Cs_{\mathrm{submin}}$  **If**
$Cs_{\min}/Cs_{\mathrm{submin}}<thresh$ (thresh=0.8 in our algorithm)    Point with $Cs_{\min}$ is regarded as the matched point  **End if**
**End if**

**End if**
For each point in $\left\{FP^{VIS}\right\}$, adopt the same matching strategyPreserve bidirectionally matched point pairs in {MP}

#### 3.4.4. Reservoir Construction and Optimal Transformation Matrix Calculation

For image sequence registration, if we only use feature points of the current frame to compute the transformation matrix, parameters may not be obtained since there may not be enough feature points to be extracted, especially when moving targets enter or leave the scene. To solve this problem, we save the matched point pairs from different frames in a reservoir. Many approaches to constructing and updating reservoirs have been proposed, for example in reference [8], a 30 or 100 frame-wide reservoir with First-In, First-Out (FIFO) update strategy is created. The disadvantage of this reservoir is that there will not be enough matched point pairs if the targets disappear during the 30 or 100 frames. And FIFO strategy may replace the better-matched point pair with worse-matched or even wrong match. In reference [14], reservoir eliminates those pairs that are regarded as persistent outliers based on the RANSAC algorithm and a proposed voting scheme. This reservoir is robust, but with the cost of high computational complexity. In reference [15], a match whose HOE matching metric is greater than the median value is considered as outliers. When a new match is generated, one of the outliers is randomly selected and replaced. This reservoir is updated by the description of the feature points, which is not applicable to our algorithm.

Based on the feature point description method in our algorithm, as introduced in Algorithm 2, we create a new reservoir updated by BIWO strategy. Once the reservoir is full, a new match is allowed to enter only when the similarity metrics meet the admission criteria.

**Algorithm 2:** Reservoir updated by BIWO strategy**Input:** Reservoir Re={(p1IR,p1VIS),(p2IR,p2VIS),…,(pnIR,pnVIS)}; new point pair $\left(p_{new}^{IR},p_{new}^{VIS}\right)$ and itssimilarity metrics ${S_{P}}^{new}$, ${S_{L}}^{new}$ and $Cs^{new}$.**Output:** Updated reservoir Re′.
**If**
n>500
Calculate the means of $S_{P}$ and $S_{L}$ of Re  **If**
${S_{P}}^{new}$ and ${S_{L}}^{new}$ are smaller than the means & $Cs^{new}$ is smaller than the maximum    Abandon the point pair with maximal Cs and replace it with the new  **End if**
**End if**
Obtain the updated reservoir Re′

The proposed reservoir updates only when the new point pair is better matched, which prevents worse-matched and wrong matches from entering it. Once the reservoir is filled with sufficient matched point pairs, the best precise parameters $\sigma^{p}$, $\theta^{p}$, $\Delta y^{p}$ and $\Delta x^{p}$ can be obtained by using RANSAC algorithm and we can get the final optimal global parameters by inverting the Equation (1):

(14) $$\{\begin{array}{l} \sigma^{f}=\sigma^{c}\times \sigma^{p}\\ \theta^{f}=\theta^{c}+\theta^{p}\\ \Delta y^{f}=\sigma^{c}\times [\cos(\theta^{c})\times \Delta y^{p}+\sin(\theta^{c})\times \Delta x^{p}]+\Delta y^{c}\\ \Delta x^{f}=\sigma^{c}\times [\cos(\theta^{c})\times \Delta x^{p}-\sin(\theta^{c})\times \Delta y^{p}]+\Delta x^{c} \end{array}$$

## 4. Experiments and Analysis

In this section, we test and analyze the performance of the proposed algorithm in the planar image sequences.

### 4.1. Dataset

For comparison with other algorithms related to our work, we choose LITIV dataset provided by reference [32] to test our algorithm. It contains nine image sequences and provides ground-truth matrices by manually selecting notable matched point pairs of moving targets in the scene. The dataset has a resolution of 240 × 320, a frame rate of 30 FPS, and lengths between 329 and 1238 frames.

### 4.2. Qualitative Results and Analysis

**Figure 8:**
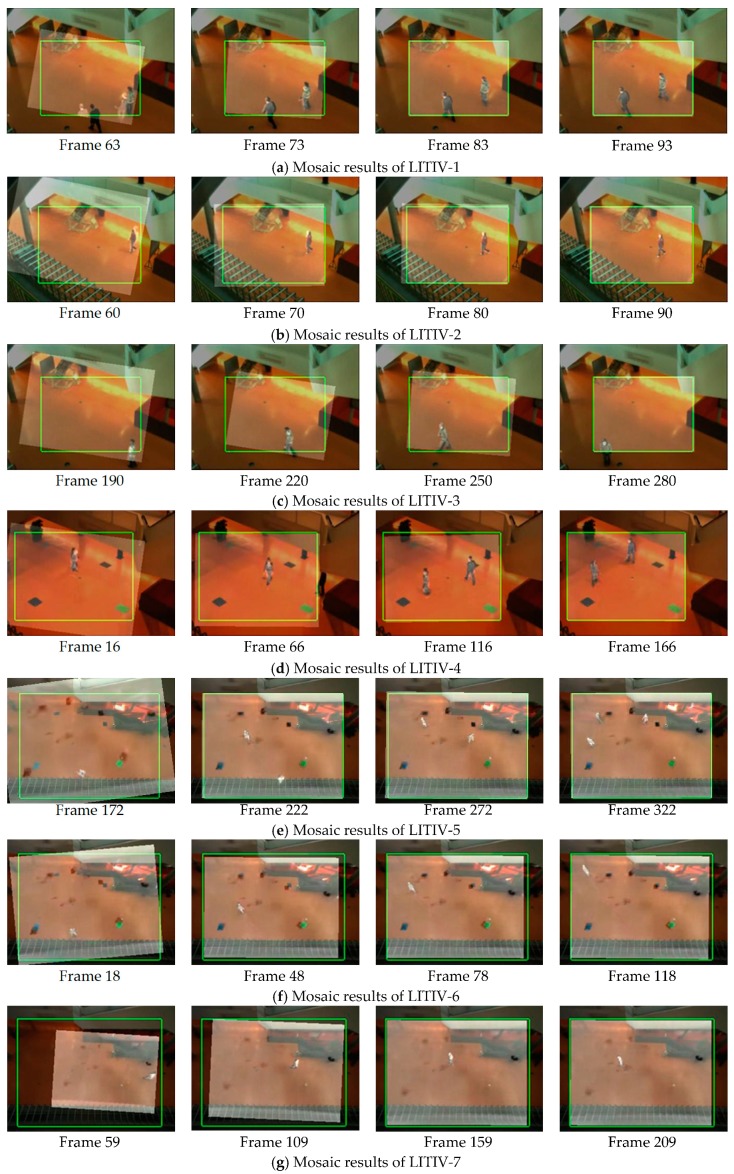

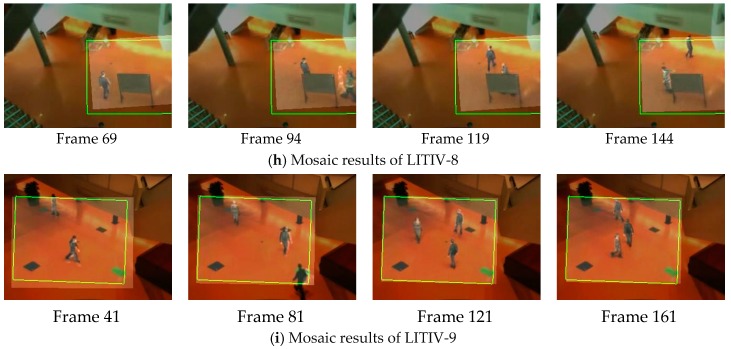
Mosaic results obtained by our registration algorithm. Registered infrared image is superimposed on the visible image. The green rectangle is the boundary transformed by the ground-truth matrix.

In the proposed algorithm, infrared image is the floating image and registered by the computed transformation matrix, while visible image is used as the reference image. To visualize the effectiveness of the proposed algorithm, in Figure 8, we show mosaic results incorporate transformed infrared images, original visible images and the ground-truth matrices of each image sequence. 

Due to the existence of coarse registration process, when targets appear in the scene, the deviation of two images is not very large, especially in LITIV-4, LITIV-6 and LITIV-9, targets have already been roughly aligned. In LITIV-6, LITIV-7, LITIV-8, and LITIV-9, the final registration matrix does not completely coincide with the ground-truth matrix. This does not mean that the proposed algorithm is unable to achieve high registration accuracy. There are two reasons for this: (1) The ground-truth matrix is obtained by manually selecting notable matched point pairs of targets, and there may be errors. In the subsequent quantitative results, we can see that the registration evaluation metric can exceed the ground-truth matrix in some frames. (2) The ground-truth matrix is unique and suitable for registration of planar scenes, but LITIV dataset does not fully satisfy the planar condition, especially in LITIV4, LITIV8, and LITIV9, moving targets in the scene are multiple, and each target has its own depth of field. We cannot directly register all the targets in the scene with the same transformation matrix. We will provide further detailed explanations for this in the following section.

### 4.3. Quantitative Results and Analysis

To quantitatively evaluate the proposed algorithm, we select two state-of-the-art algorithms for registering planar image sequences [14,15] as comparisons. Charles et al. [14] creates a framework based on shape matching, and introduces a voting scheme to define whether a matched point pair in the reservoir is persistent outliers, and eliminating it to updates the reservoir. Sun et al. [15] creates a framework combining motion and feature information. It uses motion vectors to calculate the scaling and rotation factors in coarse registration and uses HOE descriptors to describe and match the feature points in precise registration. A reservoir updated based on HOE matching metric is proposed. 

We adopt a registration evaluation metric, which is overlapping error of the foreground, defined as:

(15) $$\xi_{OE}=1-\frac{F^{VIS}\cap \Gamma (F^{IR},H)}{F^{VIS}\cup \Gamma (F^{IR},H)}$$

where $F^{VIS}$ and $F^{IR}$ are visible and infrared foregrounds respectively. $\Gamma (F^{IR},H)$ represents that infrared foreground is transformed by the transformation matrix H.

**Figure 9:**
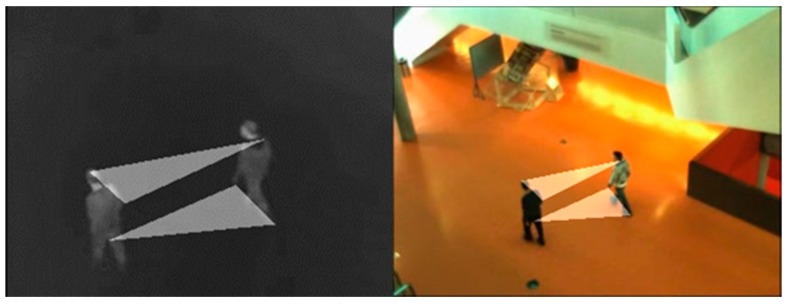
Manually connect the notable matched points of the infrared and visible targets (Frame 101, LITIV-1) to construct binary polygons.

Overlapping error of the foreground can easily be influenced by the results of the foreground extraction [14] proposed using binary polygons instead of foreground to calculate the overlapping error. Binary polygons are formed by connecting the matched points of notable parts of moving targets (heads, shoulders, palms, etc.). When the binary polygon in infrared is transformed, the overlapping error of the two polygonal regions is calculated. Figure 9 shows the binary polygons in LITIV-1.

**Figure 10:**
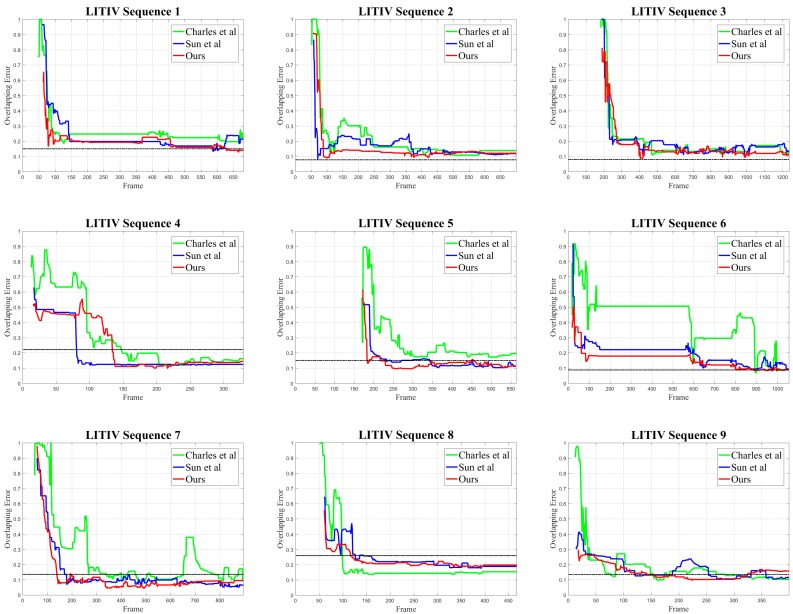
Overlapping error-time curves of nine image sequences in LITIV dataset. The red is our algorithm, the green is the algorithm of [14], and the blue is the algorithm of [15]. The dotted black represents the ground-truth matrix.

To globally show the performance of the optimal transformation matrix calculated for each frame, we plot the overlapping error-time curves and compare them with the algorithms of [14,15]. Figure 10 shows the curves of nine image sequences in LITIV dataset.

Except for LITIV-8, the overlapping error of our algorithm is lower than [14] in all image sequences, and the convergence speed is faster. [14] registers targets based on direct shape matching, which is susceptible to foreground detection results. When the moving targets in the scene enter and leave the field of view, occlusion or overlap of the targets may happen and result in inaccurate shapes of the foregrounds. Our algorithm contains coarse registration based on motion vector distribution. Even if the occlusion or target overlap occurs, identical distribution of the motion vector can be used to align the targets as long as the changes are simultaneous. LITIV-8 is special, where the spatial difference between the floating image and the reference image is large. Translation of horizontal direction is more than 100 pixels given by ground-truth. [14] directly registers the moving targets without relocation or distance constraints on feature points, so it performs better in this sequence. Despite [14] being better for a specific problem, the overall performance (for most of the LITIV dataset sequences) of our proposed algorithm is superior to [14]. For further quantitative evaluation, we give the minimum overlapping errors of each image sequence in Table 1, which reflects the optimal registration performance.

And we give the average overlapping errors of each image sequence in Table 2, which reflects the robustness of the algorithms.

Combining the curves, the minimum and the average overlapping errors, our algorithm achieves the best registration results compared to [14,15] in LITIV-1, LITIV-3, LITIV-4, LITIV-5 and LITIV-7. It should be noted that in LITIV-4, our algorithm cannot achieve lower overlapping errors for a long time. This happens because the number of connected blobs extracted by the foreground detection method is inconsistent during these frames, where there is one blob in the infrared while there are two blobs in the visible. In the feature matching process, our algorithm relies on the connected blobs to which the feature points belong. However, when the number of connected blobs returns to the same, the overlapping error begins to decrease.

Except for LITIV-4 and LITIV-8, our algorithm achieves the lowest average overlapping error compared to [14,15]. This is because the coarse registration can roughly align the targets in the scene, ensuring that the spatial deviation is not particularly large. We can see that in LITIV-1, LITIV-3, LITIV-6 and LITIV-9, our algorithm has already reached a low error at initial frames.

In all but LITIV-2 and LITIV-3 sequences, our algorithm obtains lower errors than the ground-truth, which indicates that there are potential errors in manually selecting matched point pairs to calculate ground-truth matrices, and also explains that our final matrices do not completely coincide with the ground-truth in the mosaic results.

**Figure 11:**
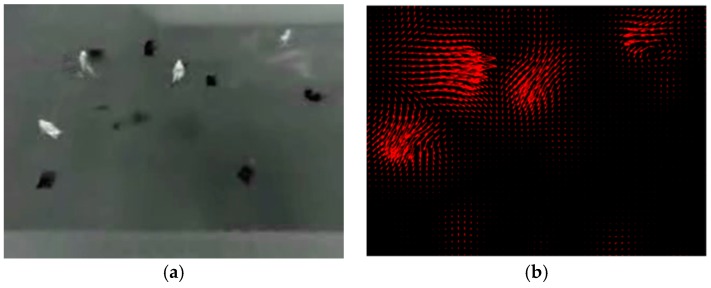
(**a**) The left is the original infrared image (Frame 328, LITIV5) and (**b**) the right is the motion vector diagram.

LITIV-5, LITIV-6 and LITIV-7 fully reflect the superiority of our algorithm. In these image sequences, we can see from the overlapping error-time curves that our algorithm keeps the lowest level for most of the time, because these sequences share the common characteristics: moving targets is far from the sensor in the scene, there is almost no overlap between moving targets and the moving directions are quite distinguishable, which are more conducive for coarse registration based on the distribution of motion vector. Figure 11 shows infrared motion vector diagram obtained from adjacent frames in LITIV-5, which has obvious discrimination.

In LITIV-1, LITIV-5, LITIV-6, LITIV-7 and LITIV-9, our algorithm converges faster than [14,15] and can achieve lower overlapping error (0.25) in a shorter time.

As for computational complexity, experiments are conducted on an Intel(R) Core (TM) i5-6500 CPU, 3.20 GHz, 16 GB RAM, Win 7 × 64, Matlab R2016a platform. Table 3 shows the average computing time of single frame of each image sequence in LITIV dataset.

In summary, the proposed registration algorithm is superior to the other two state-of-the-arts in registering infrared-visible image sequences. When moving targets in the scene are small and the motion states are distinguishable, the proposed algorithm can achieve higher registration accuracy and faster convergence speed than the other two state-of-the-art algorithms.

## 5. Conclusions

In this paper, we propose an automatic and robust infrared-visible image sequence registration algorithm through spatio-temporal association. In the coarse registration step, we use temporal motion information of the foreground, establish MVD descriptors for foreground contour points and roughly align the targets to eliminate inaccurate positioning of the feature points. In the precise registration step, we use the spatial location distribution of connected contour points to describe the detected feature points and match them under bidirectional optimal maximum strategy. BIWO strategy based on similarity metrics is created to update the reservoir and low registration overlapping errors can be obtained. Extensive evaluations well demonstrate the effectiveness of the proposed algorithm, which outperforms the other two state-of-the-arts in registering infrared-visible image sequences.

## Figures and Tables

**Table 1:** Minimum overlapping errors of each image sequence in LITIV dataset (the red represents the best results).

Sequence Pair	Ground-Truth	Ours	Sun et al.	Charles et al.
LITIV-1	0.1498	0.1297	0.1348	0.1868
LITIV-2	0.0777	0.0917	0.0825	0.1058
LITIV-3	0.0803	0.0886	0.1011	0.1083
LITIV-4	0.2213	0.0987	0.1094	0.1184
LITIV-5	0.1500	0.0956	0.1020	0.1721
LITIV-6	0.0875	0.0823	0.0831	0.0689
LITIV-7	0.1360	0.0448	0.0523	0.0909
LITIV-8	0.2596	0.1848	0.1763	0.1367
LITIV-9	0.1343	0.0954	0.0932	0.0950

**Table 2:** Average overlapping errors of each image sequence in LITIV dataset (the red represents the best results).

Sequence Pair	Ground-Truth	Ours	Sun et al.	Charles et al.
LITIV-1	0.1498	0.1933	0.2264	0.2657
LITIV-2	0.0777	0.1474	0.1617	0.2049
LITIV-3	0.0803	0.1667	0.1872	0.1932
LITIV-4	0.2213	0.2454	0.1981	0.3116
LITIV-5	0.1500	0.1339	0.1512	0.2671
LITIV-6	0.0875	0.1543	0.1902	0.4125
LITIV-7	0.1360	0.1191	0.1358	0.2573
LITIV-8	0.2596	0.2213	0.2366	0.2038
LITIV-9	0.1343	0.1503	0.1726	0.1850

**Table 3:** Average computing time of single frame.

Sequence Pair	LITIV-1	LITIV-2	LITIV-3	LITIV-4	LITIV-5	LITIV-6	LITIV-7	LITIV-8	LITIV-9
**Time(s)**	0.0615	0.1028	0.0638	0.0925	0.0781	0.0699	0.0633	0.0764	0.0733

## References

[1] Sappa A., Carvajal J., Aguilera C., Oliveira M., Romero D., Vintimilla B. (2016). Wavelet-based visible and infrared image fusion: A comparative study. Sensors.

[2] Du Q., Xu H., Ma Y., Huang J., Fan F. (2018). Fusing infrared and visible images of different resolutions via total variation model. Sensors.

[3] Zhou Z., Bo W., Sun L., Dong M. (2016). Perceptual fusion of infrared and visible images through a hybrid multi-scale decomposition with gaussian and bilateral filters. Inf. Fus..

[4] Zambotti-Villela L., Yamasaki S.C., Villarroel J.S., Alponti R.F., Silveira P.F. (2014). Novel fusion method for visible light and infrared images based on nsst-sf-pcnn. Infrared Phys. Technol..

[5] Xiao G., Yun X., Wu J. (2012). A multi-cue mean-shift target tracking approach based on fuzzified region dynamic image fusion. Sci. China Inf. Sci..

[6] Singh R., Vatsa M., Noore A. (2008). Integrated multilevel image fusion and match score fusion of visible and infrared face images for robust face recognition. Pattern Recognit..

[7] Tsagaris V., Anastassopoulos V. (2005). Fusion of visible and infrared imagery for night color vision. Displays.

[8] Sonn S., Bilodeau G.-A., Galinier P. Fast and accurate registration of visible and infrared videos. Proceedings of the IEEE Conference on Computer Vision and Pattern Recognition Workshops.

[9] Aguilera C., Barrera F., Lumbreras F., Sappa A.D., Toledo R. (2012). Multispectral image feature points. Sensors.

[10] Rui T., Zhang S.-a., Zhou Y., Jianchun X., Jian D. Registration of infrared and visible images based on improved sift. Proceedings of the 4th International Conference on Internet Multimedia Computing and Service.

[11] Zhang Y., Zhang X., Maybank S.J., Yu R. (2013). An ir and visible image sequence automatic registration method based on optical flow. Mach. Vis. Appl..

[12] Caspi Y., Simakov D., Irani M. (2006). Feature-based sequence-to-sequence matching. Int. J. Comput. Vis..

[13] Bilodeau G.-A., Torabi A., Morin F. (2011). Visible and infrared image registration using trajectories and composite foreground images. Image Vis. Comput..

[14] St-Charles P.-L., Bilodeau G.-A., Bergevin R. Online multimodal video registration based on shape matching. Proceedings of the IEEE Conference on Computer Vision and Pattern Recognition Workshops.

[15] Sun X., Xu T., Zhang J., Li X. (2017). A hierarchical framework combining motion and feature information for infrared-visible video registration. Sensors.

[16] Dalal N., Triggs B. Histograms of oriented gradients for human detection. Proceedings of the IEEE Conference on Computer Vision and Pattern Recognition.

[17] Rosten E., Porter R., Drummond T. (2010). Faster and better: A machine learning approach to corner detection. IEEE Trans. Pattern Anal. Mach. Intell..

[18] Belongie S., Malik J., Puzicha J. (2002). Shape matching and object recognition using shape contexts. IEEE Trans. Pattern Anal. Mach. Intell..

[19] Chi K.C., Tsui H.T., Tong L. (2004). Surface registration using a dynamic genetic algorithm. Pattern Recognit..

[20] Rezaei H., Shakeri M., Azadi S., Jaferzade K. Multimodality image registration utilizing ant colony algorithm. Proceedings of the 2009 2nd International Conference on Machine Vision.

[21] Thévenaz P., Unser M. (2000). Optimization of mutual information for multiresolution image registration. IEEE Trans. Image Process..

[22] Kim J., Fessler J.A. (2004). Intensity-based image registration using robust correlation coefficients. IEEE Trans. Med. Imaging.

[23] Pluim J.P., Maintz J.A., Viergever M.A. (2003). Mutual-information-based registration of medical images: A survey. IEEE Trans. Med. Imaging.

[24] Foroosh H., Zerubia J.B., Berthod M. (2002). Extension of phase correlation to subpixel registration. IEEE Trans. Image Process..

[25] Jang J., Yoo Y., Kim J., Paik J. (2015). Sensor-based auto-focusing system using multi-scale feature extraction and phase correlation matching. Sensors.

[26] Kim Y.S., Lee J.H., Ra J.B. (2008). Multi-sensor image registration based on intensity and edge orientation information. Pattern Recognit..

[27] St-Charles P.-L., Bilodeau G.-A., Bergevin R. A self-adjusting approach to change detection based on background word consensus. Proceedings of the 2015 IEEE Winter Conference on Applications of Computer Vision (WACV).

[28] Horn B.K., Schunck B.G. (1981). Determining optical flow. Artif. Intell..

[29] Bouguet J.-Y. (2001). Pyramidal implementation of the affine lucas kanade feature tracker description of the algorithm. Intel Corp..

[30] Liu T., Shen L. (2008). Fluid flow and optical flow. J. Fluid Mech..

[31] Fischler M.A., Bolles R.C. (1981). Random sample consensus: A paradigm for model fitting with applications to image analysis and automated cartography. Commun. ACM.

[32] Torabi A., Massé G., Bilodeau G.-A. (2012). An iterative integrated framework for thermal–visible image registration, sensor fusion, and people tracking for video surveillance applications. Comput. Vis. Image Understand..

